# Clinical features, epidemiology, and treatment of Shwachman-Diamond syndrome: a systematic review

**DOI:** 10.1186/s12887-023-04324-3

**Published:** 2023-10-06

**Authors:** Xue Han, Shuanglong Lu, Changjuan Gu, Zhuli Bian, Xiaotian Xie, Xiaohong Qiao

**Affiliations:** Department of Pediatrics, Tongji Hospital, Tongji University School of Medicine, 389 Xincun Road, Shanghai, 200065 China

**Keywords:** Shwachman-Diamond syndrome, Clinical feature, Exocrine pancreatic insufficiency, Bone marrow failure syndrome, Failure to thrive

## Abstract

**Background:**

Shwachman-Diamond syndrome (SDS) is an autosomal recessive disease which results in inherited bone marrow failure (IBMF) and is characterized by exocrine pancreatic dysfunction and diverse clinical phenotypes. In the present study, we reviewed the internationally published reports on SDS patients, in order to summarize the clinical features, epidemiology, and treatment of SDS.

**Methods:**

We searched the WangFang and China National Knowledge Infrastructure databases with the keywords “Shwachman-Diamond syndrome,” “SDS,” “SBDS gene” and “inherited bone marrow failure” for relevant articles published from January 2002 to October 2022. In addition, studies published from January 2002 to October 2022 were searched from the Web of Science, PubMed, and MEDLINE databases, using “Shwachman-diamond syndrome” as the keyword. Finally, one child with SDS treated in Tongji Hospital was also included.

**Results:**

The clinical features of 156 patients with SDS were summarized. The three major clinical features of SDS were found to be peripheral blood cytopenia (96.8%), exocrine pancreatic dysfunction (83.3%), and failure to thrive (83.3%). The detection rate of SDS mutations was 94.6% (125/132). Mutations in SBDS, DNAJC21, SRP54, ELF6, and ELF1 have been reported. The male-to-female ratio was approximately 1.3/1. The median age of onset was 0.16 years, but the diagnostic age lagged by a median age of 1.3 years.

**Conclusions:**

Pancreatic exocrine insufficiency and growth failure were common initial symptoms. SDS onset occurred early in childhood, and individual differences were obvious. Comprehensive collection and analysis of case-related data can help clinicians understand the clinical characteristics of SDS, which may improve early diagnosis and promote effective clinical intervention.

**Supplementary Information:**

The online version contains supplementary material available at 10.1186/s12887-023-04324-3.

## Background

Shwachman-Diamond syndrome (SDS) is an autosomal recessive disease, which represents one of the primary causes of inherited bone marrow failure syndrome (IBMFS). The most common clinical manifestations are bone marrow hematopoietic failure of varying degrees, exocrine pancreatic dysfunction, failure to thrive, bone deformity, and other congenital abnormalities. Furthermore, SDS has been associated with the development of secondary myelodysplastic syndrome (MDS), acute myeloid leukemia (AML), and other hematologic malignancies [1–3]. However, significant heterogeneity has been observed in the clinical manifestations of SDS. Although SDS is predominantly caused by mutations in the SDBS gene, a correlation between the different phenotypes of SDBS mutations and clinical manifestations has not yet been found [3–5]. As SDS is relatively rare, with an incidence rate of only 0.5–1.5/10^5^ [1, 6], most cases providing comprehensive clinical descriptions have only reported either individual cases, or small samples [7–11]. Although several case summaries have been reported in Europe and the United States in recent years, they have been limited to unilateral study results, discussing factors such as blood cell analysis, secondary tumor statistics, and the efficacy of allogeneic hematopoietic stem cell transplantation (allo-HSCT), and have failed to provide comprehensive data descriptions of pancreatic, skeletal, and other lesions [12–14]. Due to this insufficiency, to date, there remains a lack of objective and practical descriptions of the common clinical features, occurrence probability, and evolution of SDS, all of which would be essential to facilitate early diagnosis. As a result, the clinical understanding of SDS is currently lacking, and the rate of misdiagnosis is high. European and American cohort studies have shown that SDS was found to be the primary disease in many cases of patients diagnosed with secondary MDS or AML [1–3]. Furthermore, there have been many reports of missed diagnosis, resulting in a significantly delayed age of diagnosis [15, 16]. As such, it is necessary to conduct evidence-based research using the Systematic Review method commonly used in international research on rare diseases, which makes full use of shared data on online platforms [17, 18]. In the present study, we summarize the characteristics of SDS, aiming to provide evidence to improve the clinical understanding and early diagnosis rate of this poorly understood disease.

## Materials and methods

### Data sources and search strategy

**Fig. 1:**
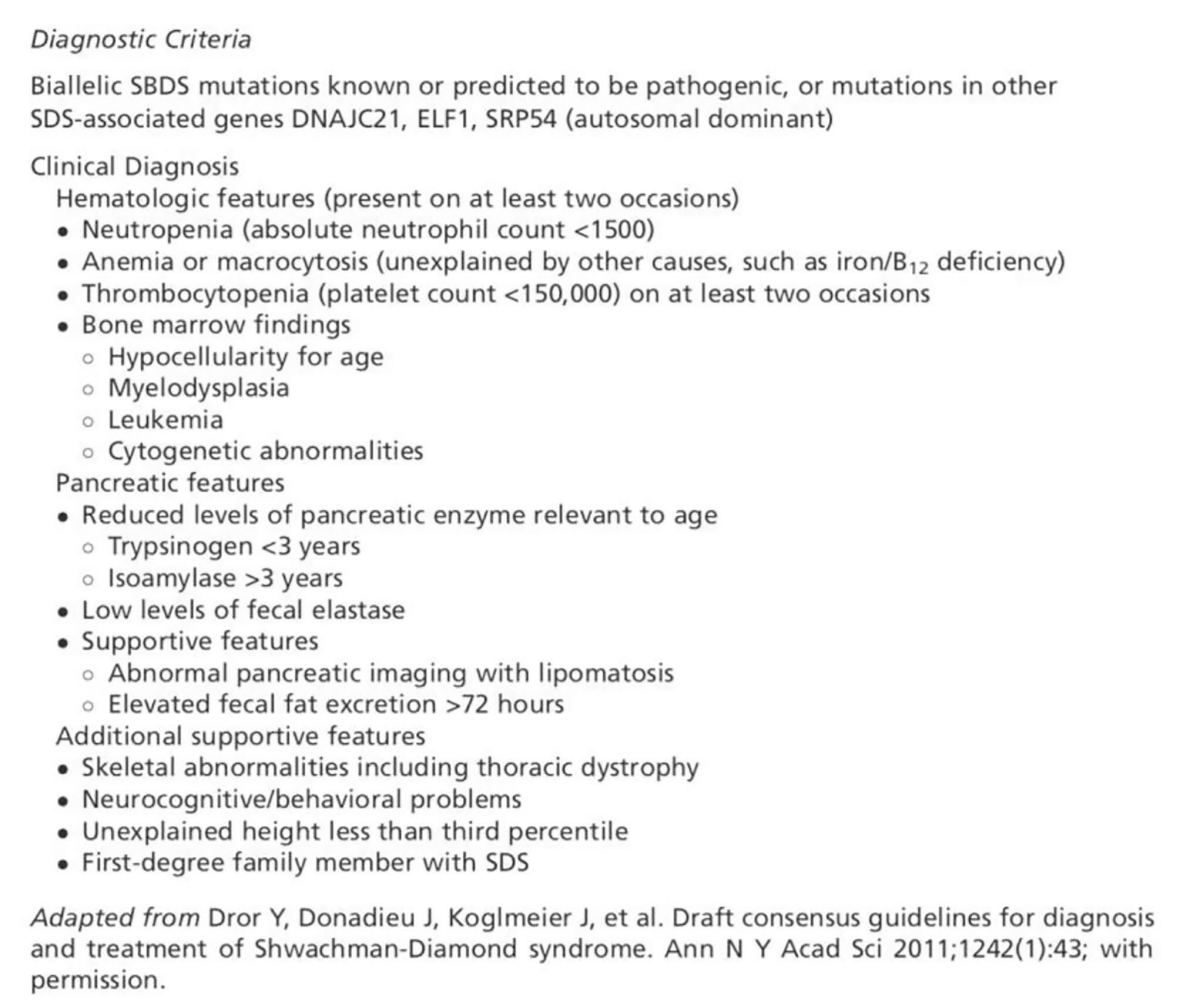
Draft consensus guidelines for diagnosis of Shwachman-Diamond syndrom

The literature was searched for articles describing cases of SDS, using the three major journal databases in China (WAN FANG DATA, VIP, and CNKI DATA) and the three major international; journal databases (Web of Science, PubMed, and MEDLINE), using the search terms “Shwachman-Diamond syndrome,” “congenital bone marrow failure” from January 2010 to October 2022. Cases were considered acceptable if they met the SDS diagnostic criteria [1, 6, 19]. As diagnostic criteria for this disease are constantly being revised, we used the latest international versions (Fig. 1). Duplicate reports were excluded. Overall, 44 cases from 23 publications in the Chinese language literature and 112 cases from 87 publications in the English language literature were included. This literature review did not require the approval of any institutional review board as it was a retrospective study and there was therefore no risk to the human subjects.

### Data abstraction and analysis

**Fig. 2:**
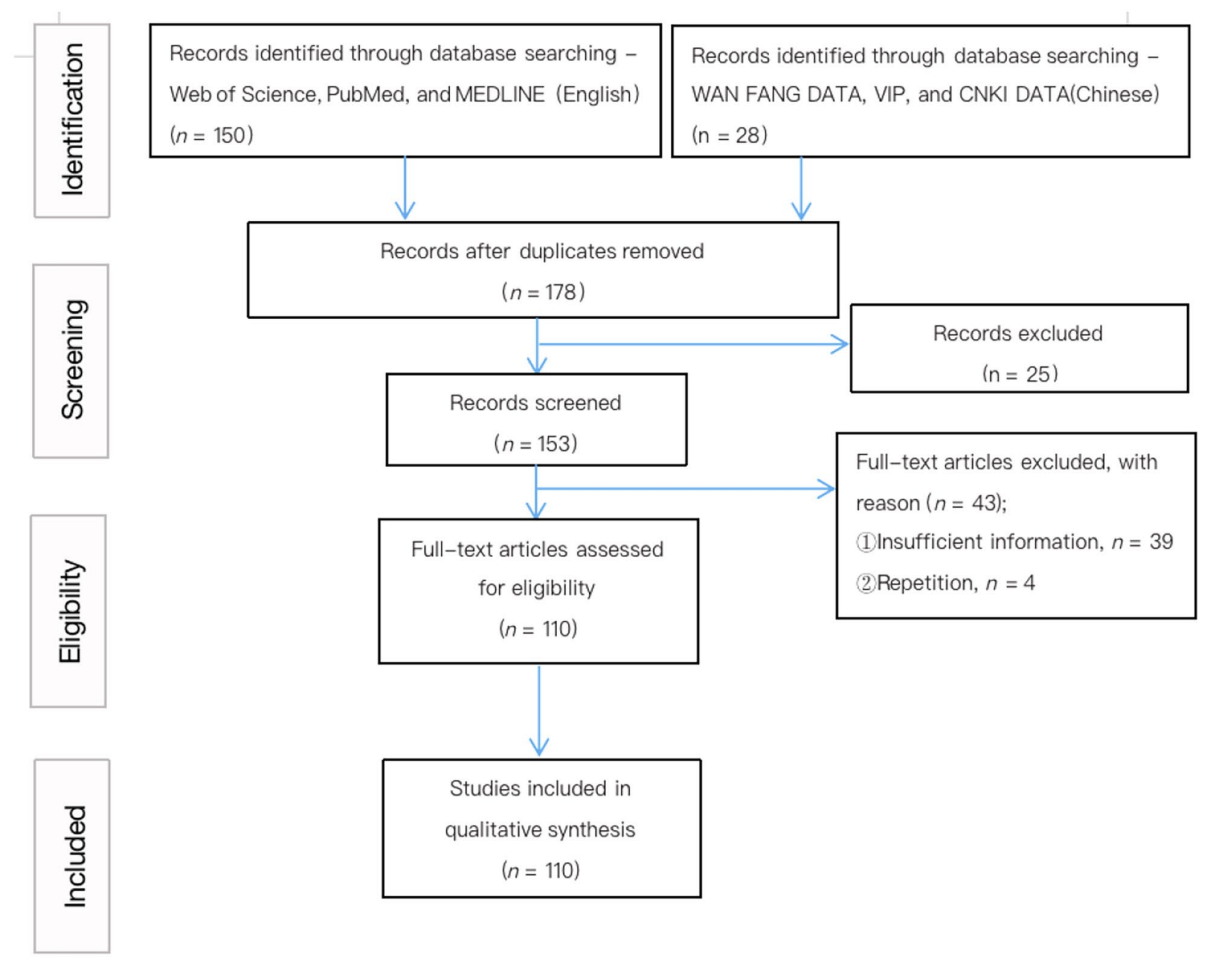
PRISMA flowchart showing the stages of the systematic review

We read 153 studies, excluding 4 duplicate studies and 39 studies with incomplete clinical data, and finally extracted the clinical data of 156 patients from 110 studies for summary and analysis (Fig. 2). The clinical features, epidemiology, disease evolution, gene mutations, diagnosis, and therapeutic measures for all cases were extensively summarized and analyzed. SPSS 24.0 statistical software was used for data processing, and measurement data were expressed as mean ± standard deviation. Data which failed to conform to a normal distribution were expressed as the median. Count data were expressed as the frequency and percentage (%), and the χ^2^ test or Fisher exact probability method were used for comparison between the two groups. Statistical significance was set at P < 0.05.

### Bias and quality assessment

Case reports and case series were uncontrolled and had an increased risk of bias in the study design. Quality assessment of the included studies was conducted using a tool validated for the systematic review of case reports/case series [20]. The results of quality assessment were not used as an exclusion criterion because of the paucity of literature in this area. All included articles received high satisfaction ratings and were published in journals with peer-review policies.

## Results

### Epidemiological characteristics of SDS in the world

The characteristics of patients included in this study are shown in Table 1. A total of 156 cases of SDS (one case in our hospital and 155 cases in the literature) were included. Of these, 87 patients were male, 67 were female, and two were of uncertain sex. The male-to-female ratio was approximately 1.3/1. The median age of onset was 0.16 years (range, 0–32 years); however, the diagnostic age lagged, with a median age of 1.3 years (range 0–49 years). Of the 84 patients for whom the gestational week was provided, 62 (73.8%,62/84) were term infants, and 22 (26.2%, 22/84) were preterm infants. The percentage of preterm infants with onset age less than 1 month (68.2%,15/22) was higher than that of full-term infants (32.3%, 20/62), and this difference was found to be significant (χ^2^ = 8.62, P = 0.03). Exocrine pancreatic insufficiency, failure to thrive, and hematological abnormalities were the first symptoms in 48 (30.8%, 48/156), 37 (23.7%, 37/156) and 36 (23.1%, 36/156) patients, respectively. Thoracic dysplasia, physical abnormalities, skin staining, abnormal liver function, sternal tenderness, and developmental delay were all reported as relatively rare initial findings.

**Table 1:** Epidemiological characteristics of SDS patients

Epidemiological characteristics	Proportion %(n/N)
Male	56.5(87/154)
Female	43.5(67/154)
Age at diagnosis (< 1year old)	37.2(54/145)
Age of onset(< 1month)	37.4(46/123)
Gestational weeks(< 37 W)	26.2(22/84)
Age of onset(< 1month) & Gestational weeks(< 37 W)	68.2(15/22)
Age of onset(< 1month) & Gestational weeks(≥ 37 W)	32.3(20/62)
Birth weight(< 2.5 kg)	45(26/58)
First symptoms	—
Exocrine pancreatic insufficiency	30.8(48/156)
Failure to thrive	23.8(37/156)
Hematological abnormalities	23.1(36/156)
Other*	—

Other*: thoracic dysplasia, physical abnormality, skin staining, abnormal liver function, sternal tenderness, developmental delay and etc.

### Symptoms of SDS

Data concerning hematological abnormalities are shown in Table 2. In total. 151 cases (96.8%,151/156) had peripheral blood cytopenia; neutropenia (88.1%,133/151), other thrombocytopenia (85 cases, 56.3%,85/151) and anemia (51.0%,77/151) accounted for more than 50%. Neutropenia was the most common hematological abnormality among SDS patients. In the enrolled patients, we identified 51 cases (33.8%, 51/151) of cytopenia in three lineages, 48 cases (31.8%, 48/151) of cytopenia in two lineages, and 46 cases (30.5%, 46/151) of cytopenia in only one lineage.

One hundred patients (64.1%,100/156) had data describing the results of bone marrow examination; however, only 81 patients (51.9%,81/156) had bone marrow hematopoietic cell data available for analysis. Among these, 20 cases (24.7%,20/81) of bone marrow examination showed malignant lesions, including 11 cases of AML (13.6%,11/81) and 9 cases of MDS (11.1%,9/81). Therefore, the results of the bone marrow test in the remaining 81 patients were used for correlation analysis with peripheral blood cytopenia.

**Table 2:** Hematological abnormalities in SDS patients

Hematological abnormality	Proportion %(n/N)
Peripheral blood routine	96.8(151/156)
Pancytopenia	33.8(51/151)
Neutropenia	88.1(133/151)
Thrombocytopenia	56.3(85/151)
Anemia	51.0(77/151)
No cytopenia	4.0(6/151)
Cytopenia in three lineages	33.8(51/151)
Cytopenia in two lineages	31.8(48/151)
Cytopenia in one lineage	30.5(46/151)
Bone marrow examination	64.1(100/156)
malignant lesions	24.7(20/81)
AML	13.6(11/81)
MDS	11.1(9/81)
Aplastic anemia	32.1(26/81)

The characteristic clinical symptoms and other abnormalities of the patients are shown in Table 3.

Regarding pancreatic lesions, 130 patients (83.3%,130/156) presented with pancreatic exocrine dysfunction; clinical manifestations included diarrhea, decreased pancreatic enzymes (lipase and/or amylase) on auxiliary examination, or fatty changes in the pancreas on imaging. Among these, 85 patients (54.5%,85/156) had a history of typical chronic diarrhea, but only 9 patients (6.9%, 9/130) developed all pancreatic lesions at the same time (chronic diarrhea + decreased pancreatic enzymes and fatty changes in the pancreas on imaging). A further 7 patients (4.5%, 7/156) showed only radiographic fat infiltration. In children with typical chronic diarrhea, routine stool examination often reveals the presence of fat droplets without red blood or pus cells, for which anti-infective treatments are ineffective. In the present study, pancreatic enzymes were detected in 74 cases, and 56.8% (42/74) of the affected children showed different degrees of pancreatic enzyme reduction. The mean lipase levels were 8.5U/L, and the mean value of amylase was 16.8U/L. Pancreatic imaging was performed in 84 patients, of whom 57.1% (48/84) showed varying degrees of fatty infiltration.

Failure to thrive, including retardation and short stature, was the predominant symptom observed in our cohort, occurring in 130 patients (83.3%, 130/156). This condition is associated with both pancreatic exocrine dysfunction and skeletal deformities; however, the corresponding incidence of skeletal deformities was > 50% (57.1%, 89/156). The primary manifestations of this latter condition include limb deformities (multiple fingers/toes or unequal lengths of the lower limbs), trunk deformities (thoracic stenosis, etc.), and head and face skeletal abnormalities (dental dysplasia, defects, etc.). Further, abnormalities such as osteoporosis, cortical thinning, and non-ossification of the metaphysis are common on imaging.

In addition, hepatopathy (50.0%,78/156) was found to be an important clinical manifestation. Other clinical manifestations include elevated alanine transaminase (ALT), aspartate aminotransferase (AST), other transaminases, and liver enlargement. ALT was 243.2 ± 176.5 U/L, and AST was 198.3 ± 118.4 U/L. Typical hepatic jaundice (total bilirubin 187 and 72 μmol/L, direct bilirubin 158 and 53 μmol/L) was observed in only two cases. None of the patients tested positive for serum-type hepatitis B or C with any diagnostic value, and transaminase levels were restored to normal after appropriate symptomatic treatment.

Other abnormalities, such as mucocutaneous lesions (rashes, papules, eczema, ulcers, bruises, skin infections, pigmentation, and ichthyosis), were identified in 43.7% (31/71) of patients. Further, ophthalmic abnormalities (photophobia, epiphora, blepharitis, keratoconjunctivitis, episcleritis, chalazion, and styes) were observed in 23.2% (13/56) of patients with SDS. Other features included hypotonia (35.6%, 16/45), splenomegaly (23.1%, 15/65), cognitive decline (23.4%,11/47), delayed language development (22.2%,10/45), cardiac anomalies (9.9%,7/71), and arthropathy (14.9%,7/47).

**Table 3:** Characteristic clinical Symptoms and other abnormalities in SDS patients

Clinical features	Proportion %(n/N)
Characteristic clinical findings	
Pancreatic exocrine dysfunction	83.3(130/156)
Chronic diarrhea	54.5(85/156)
Pancreatic enzyme reduction	56.8(42/74)
Radiographic fat infiltration	57.1(48/84)
Only radiographic fat infiltration	4.5(7/156)
Chronic diarrhea + Pancreatic enzyme reduction + Radiographic fat infiltration	6.9(9/130)
Failure to thrive	83.3(130/156)
Skeletal deformity	57.1(89/156)
Hepatopathy	50.0(78/156)
Other manifestations	
Mucocutaneous lesions	43.7(31/71)
Hypotonia	35.6(16/45)
Splenomegaly	23.1(15/65)
Ophthalmic abnormalities	23.2(13/56)
Cognitive decline	23.4(11/47)
Language development delays	22.2(10/45)
Cardiac anomalies	9.9(7/71)
Arthropathy	14.9(7/47)

### Diagnosis and examination

Genetic analyses were performed in 132 cases and revealed genetic mutations in 125 cases (94.7%,125/132). The most common mutation sites in SBDS were c.258 + 2T > C (78.0%,103/132) and c.183_184TA > CT (50.8%,67/132) resulting in amino acid changes in protein products related to K62X and C84fsX3, respectively. Other rare mutation sites included c.201 A > G, c.292-295delAAAG, c.428 C > T, c.362 A > C, c.184 A > T, c.23 A > T, c.98 A > C, c.523 C > T and C84fsX3, etc. In addition, mutations in DNAJC21, SRP54, ELF6, and ELF1 were reported in five, three, two, and one cases respectively.

We further conducted correlation analyses between mutation sites and clinical manifestations. Analysis of the two most common mutation sites (c.258 + 2T > C and c.183_184TA > CT) in 48 cases (36.4%,48/132) of both the positive expression group and 75 cases (56.8%,75/132) of other gene phenotypes showed no significant differences in sex distribution, age of onset, cytopenia, pancreatic lesions, skeletal malformations, or secondary hematological tumors. The results of gene mutation analysis are summarized in Table 4).

**Table 4:** Correlation between gene mutation sites and clinical manifestations

	c.183-184TA > CT & c.285 + 2T > C positive	Phenotypes of other genes	χ^2^	P
Case number	48	75		
Male [n (%)]	21(43.8)	33(44.0)	0.001	0.98
Median age of onset	2.4 months*	1.2 months*	—	—
Hypocytosis [n (%)]	47(97.9)	74(98.7)	—^a^	1.00
Neutropenia [n (%)]	44(91.7)	62(82.7)	—^a^	0.19
Three lineages decreased [n (%)]	12(25.0)	26(34.7)	1.281	0.26
Secondary tumor [n (%)]	3(6.3)	4(5.3)	—^a^	1.00
Pancreatic diseases [n (%)]	44(91.7)	72(96.0)	—^a^	0.43
Failure to thrive [n (%)]	40(83.3)	62(82.7)	0.009	0.92
Skeletal anomalies [n (%)]	41(85.4)	56(74.7)	2.209	0.15

* “—”: This data is not shown in the relevant foreign literature cited. —^a^ is the number of cases less than five, Fisher’s exact probability method was used, and the column of the remaining statistics is the χ^2^ value

### Analysis of data differences between countries

In our previous studies, we identified differences in the clinical manifestations of SDS patients in different geographic regions (Table 5), indicating some regional differences in such diseases. For example, the incidence of neutropenia in children with SDS in China was not significantly different from that in other Asian countries or North America (P > 0.05); however, it was statistically different from that in Europe (P < 0.05). Further, the incidence of SDS reduction in children in China was different from those in other Asian countries and North America (P < 0.05). Similarly, pancreatic lesions in other Asian countries differed significantly from those in China and North America (P < 0.05). Unfortunately, data on the age at disease onset are lacking. Further, preliminary statistics in this study did not show any correlation between the variation sites and clinical manifestations.

**Table 5:** Comparison of clinical characteristics of children with SDS in different regions of the world

Feature	China	Other countries in Asia	Europe	North America	*χ* ^*2*^	P
Case number	44	27	102	46	—	—
Male [n (%)]	25(56.8)	18(66.7)	58(56.7)	29(63.0)	1.238	0.744
Median age of onset, months	2.7	—	—	—	—	—
Median age at diagnosis, years	1.1	2.0	0.55	3.5	—	—
Neutropenia [*n* (%)]	39(88.6)	21(77.8)	23(22.5)	38(82.6)	83.421	<0.01
Three lineages decreased [*n* (%)]	12(27.2)	13(48.1)	—	7(15.2)	9.492	0.009
Secondary tumor [*n* (%)]	3(6.8)	4(14.8)	12(11.8)	3(6.5)	—^a^	0.541
Pancreatic diseases [*n* (%)]	32(72.7)	26(96.2)	—	28(77.8)	10.986	0.004
Short stature [*n* (%)]	26(59.1)	22(81.5)	60(61.2)	35(76.1)	9.743	0.021
Skeletal Anomalies [*n* (%)]	18(40.9)	15(55.6)	—	19(41.3)	1.756	0.416
Mental retardation [*n* (%)]	6(13.6)	—	25(24.5)	—	2.173	0.140
Phenotypic distribution of major genes	42	27	—	46	—	—
c.258 + 2T > C [*n* (%)]	35(83.3)	25(92.6)	—	41(89.1)	1.440	0.487
c.183-184TA > CT [*n* (%)]	26(61.9)	20(74.1)	—	31(67.4)	1.107	0.575
Other non-SBDs genes [*n* (%)]	1(2.4)	—	—	4(7.0)	—^a^	0.363

* “—”: This data is not shown in the relevant foreign literature cited. Fisher’s exact probability method was used, and the column of the remaining statistics is the χ^2^ value

### Main treatment process and prognosis

Pancreatic Enzyme Replacement: Of the 71 relevant patients, 54 (76.1%) underwent pancreatic enzyme replacement treatment, all of whom showed an improvement in symptoms. Pancreatic exocrine dysfunction is usually more pronounced in infants but can improve by preschool age.

Granulocyte colony-stimulating factor: Granulocyte colony-stimulating factor (G-CSF) is used to prevent infection. In our cohort, we identified 34 patients (64.2%, 34/53) treated with G-CSF(3–5 μg/kg/d), among which 10 cases showed a good response. The duration of neutrophil elevation varied from two weeks to four months, with some patients using neutrophil treatment for as long as nine years.

Transplantation: Some patients were diagnosed as having malignant tumors (leukemia, myelodysplastic syndrome, breast cancer, lymphoma, pancreatic duodenal carcinoma, and hepatoblastoma). Most prominently, AML, MDS, and cirrhosis were observed in 11, 9, and 2 patients, respectively. Treatment of these patients was supervised by rheumatologists and hematologists. In total, 27(36.0%,27/75) underwent some form transplantation, such as human leukocyte antigen (HLA)-identical HSCT (56.0%, 14/25), HLA-haploidentical HSCT (12.0%, 3/25), and HLA-haploidentical umbilical cord blood transplantation (20.0%, 5/25); the types of bone marrow transplants in the other 3 patients were unknown (12.0%, 3/25) (Table 6). For patients with a younger age of onset, rapid disease progression, typical clinical symptoms, and secondary malignant tumors, HLA-identical HSCT and HLA-haploidentical umbilical cord blood transplantation appeared to achieved better results.

Of the 156 included patients, 106 had available follow-up data. The median follow-up time was 2 years; 79.2% (84/106) were treated effectively, and 20.8% (22/106) died. Among the patients with positive treatment outcomes, 57 (67.9%, 57/84) did not undergo transplantation, instead receiving only symptomatic treatment, with a good prognosis; however, this could be due to the shorter follow-up period. Because SDS involves multiple systems, there are large individual differences in clinical manifestations and rates of progression. However, early diagnosis and treatment can improve the treatment efficacy and prognosis.

**Table 6:** Transplantation effect comparison

Feature	HLA-identical HSCT	HLA-haploidentical umbilical cord blood transplantation	HLA-haploidentical HSCT
Case number	14	5	3
Median age of onset, months	2.4	3.6	3.6
Median age at diagnosis, years	0.5	1.0	4.0
Neutropenia [*n* (%)]	11(78.6)	5(100.0)	3(100.0)
Three lineages decreased [*n* (%)]	6(42.9)	2(40.0)	2(66.7)
Secondary tumor [*n* (%)]	7(50.0)	1(20.0)	2(66.7)
Pancreatic diseases [*n* (%)]	12(85.7)	5(100.0)	2(66.7)
Short stature [*n* (%)]	7(50.0)	2(40.0)	1(33.3)
Skeletal Anomalies[*n* (%)]	9(64.3)	1(20.0)	2(66.7)
c.183-184TA > CT &c.258 + 2T > C[*n* (%)]	12(85.7)	5(100.0)	3(100.0)
Death[*n* (%)]	3(21.4)	0	2(66.7)
Median age of follow-up time, years	4	1	1.6

## Discussion

SDS is one of the primary causes of congenital aplastic anemia, which represents a significant health burden [21, 22]. Early diagnosis of SDS is critical because bone marrow malformations or failures often require early intervention. Although SDS is easily diagnosed in patients with characteristic skeletal malformations and clinical symptoms of pancreatic insufficiency, in patients with atypical clinical symptoms, accurate diagnosis can be challenging. Owing to the rarity of SDS, clinical data over the years are relatively comprehensive and have mostly reported on individual cases or small samples [7, 8, 23, 24], which makes it difficult to form a comprehensive description of the clinical manifestations, severity, incidence, and influencing factors of SDS with early diagnostic value. Therefore, American scholars have proposed a new version of the SDS diagnostic criteria, based on both the previous diagnostic criteria [19], and new clinical research progress .In doing so, they hoped to facilitate early clinical diagnosis, and promote extensive cooperation and data sharing by summarizing the complete clinical data of patients from all over the country, and thereby allowing continuous improvements of the description of clinical characteristics of SDS, and the subsequent clinical understanding.

In the present study, a network platform was used to share data for a systematic review to carry out evidence-based research according to the above international research progress trends, and with reference to the current international method for rare disease research [17, 18]. The main results of this systematic review can be summarized as follows: (1) A total of 156 cases of SDS who met the new diagnostic criteria for SDS were collected, allowing us to summarize the main diagnostic criteria for SDS, including gene expression, incidence of three clinical features (bone marrow failure, pancreatic lesions, and failure to thrive), and their preliminary rules. (2) Further, we identified important information that has not been reported previously; this could help to provide more valuable information about the age of onset, common initial symptoms, and the high incidence of liver damage and its clinical characteristics. (3) We further clarified the correlation between the SBDS mutation phenotype and clinical manifestations. Although the statistical results of the 156 cases were not positive, they nevertheless could be used as data to provide relevant information for international research. (4) Preliminary results indicated that there may be racial differences in the clinical characteristics of SDS. (5) Finally, we showed that for patients with slow and stable disease progression, symptomatic treatments, such as pancreatic enzyme supplementation and G-CSF, can be administered, while transplantation has a positive effect on patients with secondary malignant tumors.

In addition, this article shares the following diagnostic and treatment experiences and key tips for clinical practice: Most importantly, we showed that individuals show significant differences in clinical manifestations. Although the incidence of peripheral blood cytopenia was > 90%, the incidences of pancreatic disorders, failure to thrive, and skeletal lesions were 83.3%, 83.3%, and 57.1%, respectively. Further, although exocrine pancreatic insufficiency and failure to thrive were the most common initial symptoms, they occurred in only approximately 30.8% and 23.7% of patients, respectively. Only 6.9% (9 cases) of the patients had chronic diarrhea, decreased pancreatic enzyme levels, and imaging pancreatic steatosis, and only 32.1% (26 patients) of the patients met the diagnostic criteria for acquired aplastic anemia. Further, this analysis showed that some of the main clinical features (failure to thrive, skeletal lesions, and secondary tumors) are inevitably delayed, making early diagnosis difficult. Overall, these results show that the early manifestations of SDS are often atypical and require clinical attention.

As SDS is a rare disease, its diagnosis and reporting are heavily dependent on the level of local medical care and the knowledge of physicians about the disease. As such, there were some regional differences in the reports, and the sample size in this study was smaller than the actual sample size. Owing to the small sample size and shared network data, the conclusions obtained may contain some errors, and may not be a perfect reflection of the actual situation.

This systematic review had some limitations which should be discussed. Because the data was extracted from prior studies, it is possible that there may have been some differences from the actual data, which may have depended on the local doctors’ understanding of this disease. Further, as with any systematic review, there is a possibility of publication bias. Finally, as the cases were extracted from reports from various countries, although the reported diagnoses were in line with the diagnostic criteria at the time, the diagnostic criteria may have been revised in accordance with increasing international awareness of the disease. As such, there may have been some differences between the reported and actual cases.

As mentioned above, there may have been some discrepancies between the information used in this study and actual data, largely due to the lack of knowledge about SDS among physicians. Nevertheless, we hope that the data in this paper will help improve the clinical understanding of SDS, and promote the early diagnosis rate. Further, we hope that other clinicians and physicians will continue to accumulate clinical data, improve the description of SDS characteristics, and promote clinical attention and research on rare diseases. Finally, we hope that the systematic review and evidence-based research through network data sharing adopted in this study can provide an important reference for rare disease research.

## Conclusions

SDS is a complex disease characterized by multiple abnormalities. This condition is associated with an early age of onset, congenital malformations, and exocrine pancreatic insufficiency. We and others believe that the diagnosis and treatment of SDS can be improved through the collaboration of a multidisciplinary team, comprising pediatricians, hematologists, and oncologists. This study presents several important findings regarding the management of SDS patients. Most importantly, we showed that delayed diagnosis may occur due to an insufficient understanding of SDS. Regarding treatment, some patients with AML and MDS underwent bone marrow transplantation. Further, some SDS patients received immunosuppressive therapy with better results, which seems to confirm the belief of some scholars that SDS may be an autoimmune inflammatory disease. We hope that the future application of second-generation sequencing will improve our understanding of this disease.

### Electronic supplementary material

Below is the link to the electronic supplementary material.

Supplementary Material 1

## Data Availability

Data sharing is not applicable to this article as no new data were created or analyzed in this study. Data are available upon request to the corresponding author (Xiaohong Qiao).
