# Supplementary figures and images for: Clinical features, epidemiology, and treatment of Shwachman-Diamond syndrome: a systematic review
Source: BMC Pediatr. 2023 Oct 6;23:503. doi: 10.1186/s12887-023-04324-3 (PMC10557232; doi:10.1186/s12887-023-04324-3)

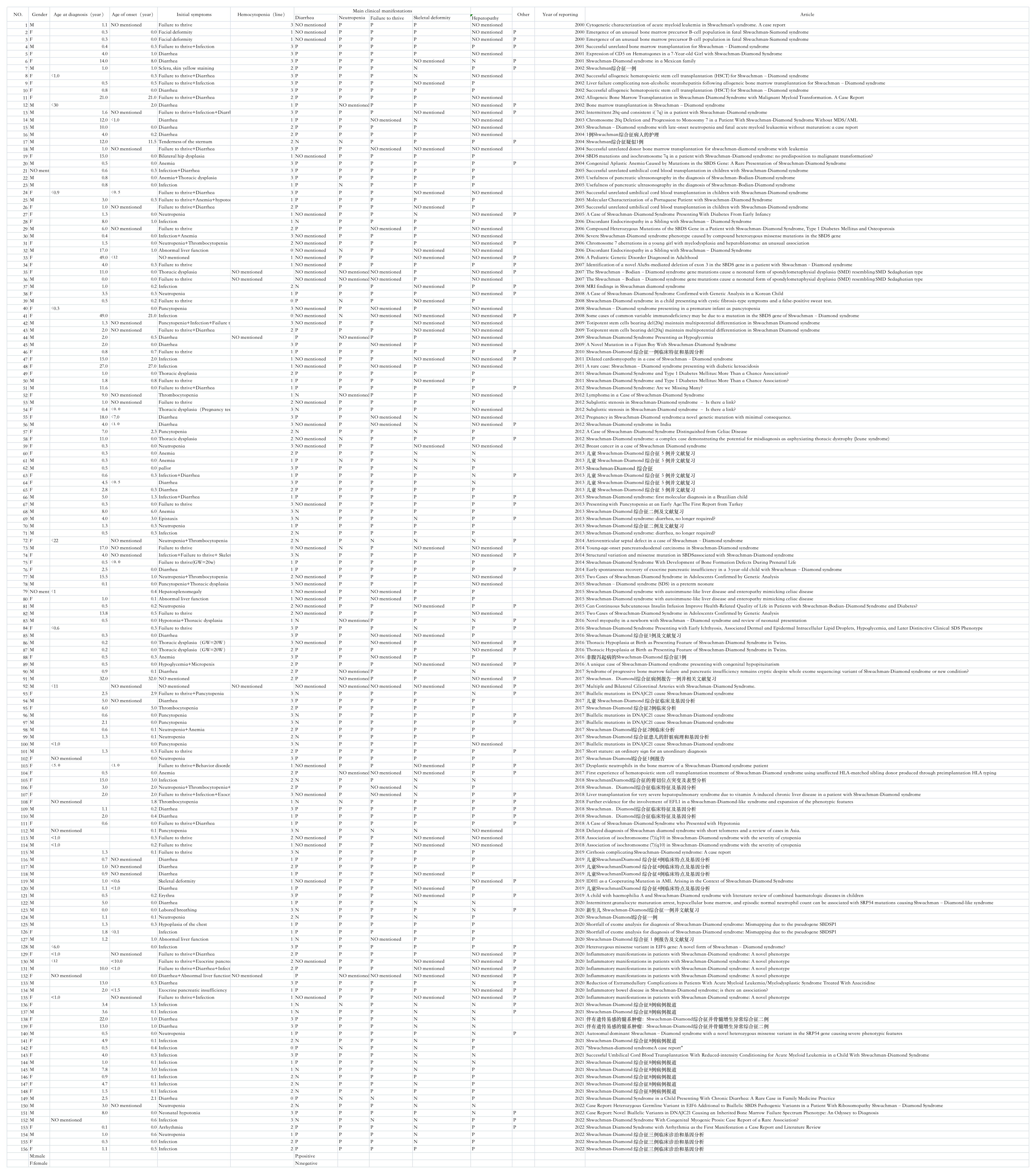

Supplement: Supplementary file 1 — Supplementary Material 1 [file 12887_2023_4324_MOESM1_ESM.png]
